# GnRH antagonist administration to postpone a weekend intrauterine insemination: a large cohort study from a public center

**DOI:** 10.1186/s12958-016-0187-4

**Published:** 2016-09-02

**Authors:** J. Gobernado, C. Alvarez-Colomo, L. Rodriguez-Tabernero, L. Barrero, J. M. F. Fernández-Gómez, J. Schneider

**Affiliations:** 1 Valladolid University Clinic, Valladolid, Spain; 2 Department of Gynecology, Valladolid University Medical School, Valladolid, Spain; 3 Facultad de Medicina, Universidad de Valladolid, Avenida de Ramón y Cajal, 47003 Valladolid, Spain

## Abstract

**Background:**

In Spanish public hospital Reproduction Units it is very problematic to perform programmed intrauterine insemination (IUI) on weekends, if indicated. Small previous pilot studies suggest that using a GnRH antagonist to avoid an LH weekend surge would allow to perform IUI on the following Monday, not impairing the expected pregnancy rate.

**Methods:**

Between 1st January 2007 and 31st December 2015, 4.782 intrauterine inseminations were performed at Valladolid University Clinic, Spain, corresponding to 1.650 women. Of them, 911, corresponding to 695 women, should ideally have been performed during the weekend. If it happened that a member of the Reproduction Unit was on duty during that particular weekend, the standard protocol was not interrupted, and the IUI performed as planned (control group, 685 IUIs). If the former was not the case, the weekend gap was bridged by administering 0.25 mg GnRH antagonist (GnRHa). Ovulation was induced by means of 250 ug recombinant HCG (rHCG) 36 h prior to IUI on the following Monday (study group, 226 IUIs).

**Results:**

There were no differences in the clinical pregnancy rate (13.7 cc vs. 16.2 %, *p* = 0.371) or in the ongoing pregnancy rate between groups (11.9 % vs. 14.9 %, *p* = 0.271). The multiple pregnancy rate was also comparable in both groups (14.7 % vs. 18.5 %, *p* = 0.77).

**Conclusions:**

Women with a planned IUI which cannot be performed at the ideal date can be offered postponement for two days with the support of GnRHa treatment, with results that are not inferior to those expected applying the regular protocol.

## Background

Since their introduction into assisted reproduction treatment schedules, both GnRH agonists [1] and antagonists [2] now routinely form part, accompanied by controlled ovarian hyperstimulation (COH), of “in vitro” fertilization (IVF) protocols, but much less so of intrauterine insemination (IUI) protocols. Accumulation of scientific evidence in their favor [3, 4] has led to their being recommended for IVF by most scientific Fertility Societies [5]. The main advantages attributed to the use of both GnRH agonists or antagonists concomitant to COH is the suppression of an unwanted LH peak which might lead to follicle luteinization at the end of ovarian stimulation. The latter is associated with a significantly worse outcome of IVF cycles, or their cancellation, if premature luteinization is suspected, with the accompanying frustration and cost increase for the patient [6–8]. In Reproduction Units of Spanish public hospitals it is very problematic, for logistical reasons, to perform IUI on weekends, if this were to be the ideal point at which IUI should take place, i.e., 36 h after reaching optimal follicle growth (2–3 follicles > 17 mm) and hCG administration. In order to avoid this organizational drawback, Matorras et al. [9] proposed in 2006 what they called a “weekend-free protocol” (and more appropriately should have been called a free-weekend protocol). It consisted in using a GnRH antagonist to avoid a very possible LH weekend surge, according to measured follicle size and estradiol levels on the previous workdays, and thus be able to perform the IUI on the following Monday. Gonadotropin Releasing Hormone antagonists have the advantage of their flexibility of use, since they can be administered at any time of the follicular phase. They suppress LH levels, and less so FSH levels, shortly after (6 h), so that, in theory, unwanted ovulation may be delayed. The results of the study by Matorras et al. [9] and of a similar, randomized one by Checa et al. [10] were extremely encouraging, in spite of their relatively small sample size. This led us to adopt their proposed protocol in 2007, and we are presenting our results after 9 years of continued use in a considerably larger patient cohort. The implementation of the protocol depended on whether a member of the Reproduction Unit was on shift during the weekend or not. This could not be chosen either by the patient or the physician, because the weekend 24-h shifts are programmed on a monthly basis, taking into account the available personnel at the time. Thus, it is the nearest thing to a randomized study that can be attained in practice in a public hospital in our country, where weekend shifts must be evenly distributed among all the members of the Department, regardless of their particular interests or those of their patients. The situation is entirely different in private Reproduction Centers, where the activity is tailored to fit the needs of the attended women, so that if an IUI has ideally to take place during a weekend or a holiday, schedules are arranged so that it does indeed take place.

## Methods

This is a retrospective study, encompassing the experience at our center between 1st January 2007 and 31st December 2015.

During the study period, a total 4.782 intrauterine inseminations (IUIs) were performed at Valladolid University Clinic, Spain, corresponding to 1.650 women. Of them, 911 IUIs, corresponding to 695 women, should ideally have been performed during the weekend, because periovulatory follicles ≥ 16 mm were ultrasonographically detected on Thursday or Friday.

If it happened that a member of the Reproduction Unit of our Center was on duty during that particular weekend, the standard protocol was not interrupted, and the IUI performed as planned (control group, 685 IUIs). If the former was not the case, the weekend gap was bridged by administering 0.25 mg Gonadotropin Relasing Hormone antagonist (GnRHa) daily in order to avoid an unwanted LH surge and premature ovulation. Planned ovulation was induced by means of the ambulatory administration of 250 ug recombinant Human Chorionic Gonadotropin (rHCG) 36 h prior to the planned IUI, which took place on the following Monday (study group, 226 IUIs). Inclusion criteria into the IUI program of our Center are: age less than 38, tubal permeability assessed by means of hysterosalpingography and total normal mobile sperm (TNMS) sperm count above 5 million/ml.

The detailed standard protocol for treatments was as follows: Ovarian stimulation was performed by means of 50-100 IU/day recombinant Follicle-Stimulating Hormone (rFSH) in all cases, until the desired follicular size was attained. Ovarian stimulation was performed with any of the two marketed recombinant FSH (rFSH) presentations (Puregon, MSD, or Gonal-F, Merck). The standard FSH dose was 75 IU/day, which was adjusted according to following criteria: patients with age <30 and/or a follicular count >10 were treated with 50 IU/day; conversely, patients with age >35 and/or an immediately previous monofollicular cycle were treated with 100 IU/day. The first control was performed at day 5–7 of treatment, and the FSH schedule adjusted if necessary. was used indifferently. When 1–3 follicles with a diameter ≥ 17 mm were detected, ovulation was induced by administering 250 ug recombinant HCG (Ovitrelle, Merck). When more than 3 follicles with a diameter ≥ 17 mm or more than 5 follicles with a diameter ≥ 14 mm developed, the cycle was aborted. In those cycles where the weekend was bridged by administering a GnRH antagonist (Cetrorelix (Cetrotide, Merck) or Ganirelix (Orgalutran, MSD) 0,25 mg), rFSH treatment was maintained until the induction of ovulation by means of rHCG (Ovitrelle, Merck). Insemination was performed 34–36 h later.

Semen capacitation was performed by means of the density gradient method. Beta-HCG levels were determined 14–16 days after IUI, and if they were elevated, a vaginal ultrasound examination was performed at 5 weeks post-IUI, in order to ascertain embryo implantation.

The variables included into the study were: clinical and ongoing pregnancy, total rFSH dose, number of 14–17 mm follicles, endometrial thickness, mobile sperm count after capacitation, and total expense on medication (taking into account prices in Spain on 31st December 2015).

Clinical pregnancy was defined by beta-HCG elevation plus ultrasonographic evidence of a gestational sac, intrauterine or extrauterine, with or without detectable cardiac activity. Ongoing pregnancy was defined by an intrauterine normally evolving pregnancy.

### Statistics

Data were processed using the Statgraphics XVII statistical package (Statpoint Technologies Inc., Warrenton, VA, U.S.A.).

For qualitative variables, we studied the frequency distribution. For continuous variables, we first calculated their adjustment to a normal distribution by means of the Kolmogorov-Smirnov test. The only studied variable to follow a normal distribution was treatment cost. In this case, the mean with a 95 % confidence interval and standard deviation was calculated. The remaining continuous variables, which did not follow a normal distribution, were compared by means of Mann–Whitney’s test, and results expressed as means, together with the corresponding standard deviation. Qualitative variables were compared by means of contingency tables and the Chi-square test. Results were considered significant if the *p*-value was < 0.05.

## Results

There was no difference in patient age between groups. Owing to the protracted date of insemination, there was a significant difference between the study and control group in the amount of rFSH employed and the duration of ovarian stimulation, which were both higher for the study group (*p* < 0.001). The follicle count was slightly, but significantly (*p* = 0.031) higher in the study group (1.4 ± 0.9 vs. 1.2 ± 1.2). There were no differences in either endometrial thickness or mobile sperm count between both cohorts of patients and partners, respectively. However, the cost of medication was significantly higher for the study group (*p* < 0.001). Most importantly, there were no statistically significant differences in the clinical pregnancy rate (13.7 % vs. 16.2 %, *p* = 0.371) or in the ongoing pregnancy rate between groups (11.9 % vs. 14.9 %, *p* = 0.271). All the aforementioned results are summarized in Table 1.

**Table 1:** Results of IUI in the study group receiving GnRHa to bridge the weekend (*n* = 226), with IUI on Monday, and the control group (*n* = 685), with IUI during the weekend as scheduled

	Study group	Control group	*p*-value
Age (years)	33.4 ± 2.8	33.2 ± 3.1	0.614
total rFSH per cycle (IU)	696.2 ± 295.5	596.5 ± 304.4	<0.001
Duration of stimulation (days)	10.3 ± 2.8	8.4 ± 2.8	<0.001
n follicles 14–17 mm	1.4 ± 0.9	1.2 ± 1.2	0.031
Endometrial thickness (mm)	9.0 ± 2.1	9.2 ± 3.3	0.554
Mobile sperm count (million)	14.5 ± 12.5	15.6 ± 13.3	0.230
Medication expenses (€):	452.72 ± 157.60	303.22 ± 132.93	<0.001
Clinical pregnancy (%)	31 (13.7 %)	111 (16.2 %)	0.371
Clinical pregnancy OR	0.78 (0.49–1.22)		
Ongoing pregnancy (%):	27 (11.9 %)	102 (14.9 %)	0.271
Ongoing pregnancy OR	0.82 (0.53–1.26)		

The multiple pregnancy rate was similar in both groups (14.7 % vs. 18.5 %, *p* = 0.77), (Table 2).

**Table 2:** Single and multiple ongoing pregnancy rate in the study and control groups

	Study group	Control group
Singleton (%)	22 (81.5 %)	87 (85,3 %)
Twins (%)	4 (14.8 %)	12 (11,8 %)
Triplets (%)	1 (3.7 %)	3 (2,9 %)
Chi-square (*p*-value)	0.77	

## Discussion

From the results of our study, it appears that bridging the weekend gap by means of GnRHa in order to minimize the occurrence of an LH surge and subsequent spontaneous ovulation, and to postpone a planned IUI until the following Monday, results in a similar pregnancy rate. Our results are in agreement with those of two previous pilot studies [9, 10], one of them randomized, but with a very low patient number. Both were initially reassuring, but there always remained a fear: that a larger series might reveal that GnRHa administration, although successful for postponing ovulation, would not avoid premature luteinization of mature follicles, and this would ultimately result in a lower pregnancy rate. Our results, with a much larger studied event number, and a considerable random effect in the assignation of women to either the study or the control group, reinforces the reassuring message provided by the studies of Matorras et al. [9] and Checa et al. [10]. Furthermore, the only initial significant difference between groups in our study was the periovulatory follicle count, which was higher in the study group receiving GnRHa, a finding that could potentially worsen the chances of successfully bridging the weekend gap until the moment of IUI. In spite of it, the pregnancy rates were still comparable. Moreover, another potential danger associated with the use of GnRHa, namely an increase of multiple pregnancies, has not taken place in our study. Although there were more multiple pregnancies in the study group compared to controls (18.5 % vs. 14.7 %), the difference did not reach statistical significance. In any case, it was far lower than the one reported in the two previous cited studies by Checa et al. (42.0 %) and Matorras et al. (42.9 %) [9, 10]. The IUI protocol of our Reproduction Unit mandates for cancellation of the cycle if more than three follicles with a diameter above 16 mm or more than five with a diameter above 13 mm are detected, and this may explain our relatively low multiple pregnancy rate.

It might be argued, finally, that the use of additional medication for postponing IUI until after the weekend carries with it a considerable increase in cost, which is true, as we have shown. However, it must be borne in mind that this increase in cost is almost exactly half the cost of a completely new cycle, which is offered if the planned one is interrupted due to its unhappy coincidence with a weekend. All this considered, the final result is a net saving of expenses. Furthermore, there are other, less quantifiable gains associated with the use of the study protocol, such as the avoidance of the frustration for the patient associated with the interruption of a fertilization cycle.

A final, fundamental doubt remains: would we obtain similar results, if we were to bridge the weekend gap without the use of any additional medication, speculating on the survival of oocytes after an eventual spontaneous ovulation? The answer to this question would be only definitely provided by a randomized study, in which the study group would be made up of women accepting not to receive a medication which has proven successful in previous studies, including this one. Such a scenario, however desirable, is difficult to envisage in practice. Until such a study can be carried out, our confirmatory results of the previous pilot studies addressing the same issue, this time carried out on a considerably larger population, are reassuring.

## Conclusions

Our data, together with those of the two previous pilot studies, strongly suggest that women with a planned IUI which cannot be performed at the ideal date for whatever reason, can be offered postponement for two days with the support of GnRHa treatment, with results that are not inferior to the ones expected for the regular protocol.

## Abbreviations

FSH: Follicle-stimulating hormone
GnRHa: Gonadotropin Relasing hormone antagonist
IU: International units
IUI: Intrauterine insemination
LH: Luteinizing hormone
rHCG: recombinant Human chorionic gonadotropin
TNMS: Total normal mobile sperm
